# Syphilis in the Middle Ages and in Modern Times

**Published:** 1898-07

**Authors:** 


Syphilis in the Middle Ages and in Modern Times. By Dr. F. Buret. Paris, France. Translated from the French, with notes, by A. H. Ohmañ-Dumesnil, M. D. Professor of Dermatology and Syphilology in the Marion Sims College of Medicine and Surgery, St. Louis, M. D., being volume II and III of "Syphilis Today and Among the Ancients." Published by The F. A. Davis Co., Philadelphia.

This curious and instructive work has been written to prove the thesis, that syphilis, instead of being a modern disease, orignating or at least being first observed, about the time of the French invasion of Italy, in 1494, is more ancient than recorded history. Whether he has succeeded in establishing the truth of his theory or not (and we think he has), he has unearthed many curious and valuable contributions to medical literature. It is also demonstrated in the course of his work that there has existed a contiuous medical liturature, from the remotest antiquity, on down through the dark ages, to the present. The work is the product of much erudition and remarkable research. J.

